# Excision of a Tuberculous Vesicula Seminalis

**Published:** 1900-01-20

**Authors:** 


### EXCISION OF A TUBERCULOUS VESICULA SEMINALIS.

At the last meeting of the Medical Society Mr. Mansell Moullin read a paper on excision of the vesicula seminalis, based upon two cases in which he had performed the operation, in conjunction with that of castration, for tuberculous disease extending upwards from the epididymis. Both the patients were young adults. In each case the testis was removed through an inguinal or inguino-scrotal incision, and the cord was divided as high up as possible. The patient was then placed in the lithotomy position, and the under surface of the prostate and the base of the bladder were exposed. The vesicula seminalis and the ejaculatory duct were then dissected out. In one case, when this had been done, a small portion of the vas lying between the two incisions was left, but in the other case two ligatures were placed upon the cord in the groin, one upon the vas deferens itself and the other upon all the rest, and then the whole remaining portion of the vas was removed through the perineal opening. The wounds were packed with iodoform gauze, and the external sphincter was sutured to the centrum tendineum. One of the cases healed up without any trouble, but in the other a small urinary fistula remained for some months, a few drops coming through on micturition. In this case the ejaculatory duct had been torn out from the urethra. Mr. Moullin strongly advocated the removal of the vesicula in every case of tuberculous epididymitis in which it could be felt distinctly through the rectum, the object being to clear out the whole of the disease, and thus give the patient a chance before the bladder became hopelessly involved. In the discussion which followed it was pointed out that the operation was by no means an easy one, and that an inexperienced operator might find himself in grave difficulties.

